# Germline Predisposition in Pediatric Central Nervous System Tumors: Insights from a Multigene Panel Study

**DOI:** 10.32604/or.2026.079120

**Published:** 2026-05-21

**Authors:** Meerim Park, Seungman Park, Ensel Oh, Jongmun Choi, Mi Mi Kwon, Seog-Yun Park, Jun Ah Lee, Hyeon Jin Park

**Affiliations:** 1 Department of Pediatrics, Center for Pediatric Cancer, National Cancer Center, Goyang, Republic of Korea; 2 Department of Laboratory Medicine, National Cancer Center, Goyang, Republic of Korea; 3 Geninus Inc., BI Center, Seoul, Republic of Korea; 4 NGS Research Center, Seegene Medical Foundation, Seoul, Republic of Korea; 5 Department of Pathology, National Cancer Center, Goyang, Republic of Korea

**Keywords:** Central nervous system (CNS) tumor, children, adolescents, and young adults (AYAs), germline variants, next-generation sequencing

## Abstract

**Objectives:** Germline variants in cancer predisposition genes have been increasingly recognized in pediatric cancers. However, their spectrum in East Asian children with central nervous system (CNS) tumors remains insufficiently defined. This study investigated the prevalence and clinical significance of pathogenic or likely pathogenic (P/LP) germline mutations in Korean children, adolescents, and young adults (AYAs) with CNS tumors. **Methods:** We performed targeted next-generation sequencing of 358 cancer-associated genes using peripheral blood DNA from 108 patients. Germline variants were classified according to ACMG/AMP guidelines and curated using ClinVar and relevant literature. **Results:** Among 108 patients, 17 (15.7%) carried P/LP germline variants. The median age at diagnosis was 7.7 years (range, 1.0–24.0), and 64.7% were male. P/LP variants were most frequent in other CNS tumors (4/11, 36.4%), including 2 glioneuronal tumors, 1 schwannoma, 1 atypical teratoid rhabdoid tumor (ATRT), and gliomas (9/32, 28.1%), followed by medulloblastomas (3/31, 9.7%). In gliomas, P/LP variants in MLH1, NF1, MUTYH, PALB2, PMS2, FANCM, and TP53 were observed, while medulloblastomas carried alterations in SUFU, BRIP1, and FANCI. SMARCB1 variant was found in ATRT. Among 25 patients with intracranial germ cell tumors, only a single case carried a P/LP germline variant, identified in FANCI. **Conclusion:** Germline P/LP mutations were identified in 15.7% of Korean children and AYAs with CNS tumors, most commonly in gliomas and other CNS tumors. Our findings highlight the molecular heterogeneity of germline predisposition in CNS tumors and emphasize the importance of germline testing for risk assessment and surveillance.

## 1 Introduction

Pediatric central nervous system (CNS) tumors are the second most common pediatric malignancy after leukemia and form a heterogeneous group of tumors. Pathogenic/likely pathogenic (P/LP) germline mutations in cancer predisposition genes have been shown to be present in 8.5% of children with newly diagnosed cancer [1]. Germline variants in cancer predisposition genes are found in approximately 8–23% of pediatric CNS tumor patients, depending on the cohort and gene panel used [2,3,4,5]. Germline variants are identified more frequently in particular tumor types such as atypical teratoid/rhabdoid tumors (ATRTs) and choroid plexus carcinomas [5]. However, the genetic background of predisposition for the development of pediatric CNS tumors has been insufficiently characterized compared to other types of childhood cancers. Previously, germline genetic alterations were considered only for patients with a typical family history or clinical features of a single-gene disorder. Children with cancer without a typical family history have been considered to be less likely to have hereditary genetic alterations.

The contribution of germline genetic susceptibility to pediatric CNS tumors remains insufficiently defined, yet the identification of germline alterations is increasingly recognized as an essential component of both short- and long-term patient management. With the expanding use of next-generation sequencing (NGS) and continuous updates to genomic databases, the detection and clinical interpretation of P/LP germline variants have become increasingly important. However, current knowledge is largely based on studies in Western populations [2]. The large-scale, population-based germline analyses in East Asian children are scarce. Considering potential ethnic differences in variant spectra and allele frequencies, delineating the germline landscape of Korean pediatric CNS tumors is crucial for refining genetic counseling, risk stratification, and precision care.

Therefore, this study aimed to determine the prevalence and clinical significance of germline cancer-related gene alterations in Korean children, adolescents, and young adults (AYAs) with CNS tumors.

## 2 Materials and Methods

### 2.1 Patients

This study enrolled patients who visited the Center for Pediatric Cancer, National Cancer Center, Republic of Korea, between June 2021 and October 2024, regardless of the timing of their initial CNS tumor diagnosis. The cohort therefore includes both newly diagnosed patients and those in long-term follow-up after treatment. Patients were not pre-selected based on clinical suspicion of cancer predisposition syndrome, family history, or any other genetic criteria.

Inclusion criteria were: (1) age at diagnosis < 25 years, (2) a pathologically confirmed CNS tumor, and (3) availability of a peripheral blood sample of adequate quality for germline genetic testing. Exclusion criteria were: (1) insufficient DNA quality or quantity to meet minimum sequencing requirements, or (2) withdrawal of informed consent.

A total of 108 patients met these criteria and comprised the final study cohort. Clinical information, including family and medical histories, diagnoses, treatment history, and comorbidities, was retrospectively collected from the medical records. This study was approved by the Institutional Review Board of the National Cancer Center (NCC2021-0140) and was conducted in accordance with the Declaration of Helsinki. Written informed consent was obtained from all participants or their legal guardians.

### 2.2 Genetic Analysis

Genomic DNA was extracted from 400 μL of EDTA-anticoagulated whole blood using the QIAamp DNA Mini Kit (QIAGEN, Hilden, Germany; Catalog No. 51306) with doubled reagent volumes. A total of 100 ng DNA was purified using a silica membrane spin column and eluted in Buffer AE. DNA concentration and purity were assessed using a Qubit™ 4.0 fluorometer and NanoDrop One spectrophotometer, respectively. Samples were stored at −20°C until use. Libraries were prepared using the commercial panel, PedSCAN^TM^ (Geninus Inc., Seoul, Republic of Korea), following the manufacturer’s standard protocol. PedSCAN^TM^ is designed to detect single nucleotide variants (SNVs), small insertions/deletions (INDELs), and copy number variations (CNVs) in 358 genes related to pediatric cancers (Supplementary Table S1). Sequencing was performed on NextSeq 550 or NovaSeq 6000 platforms (Illumina, San Diego, CA, USA) with 2 × 150 bp paired-end reads. The mean sequencing depth was approximately 1400× and uniformity (the percentage of target regions with coverage exceeding 50% of the mean depth) was 97%.

Raw sequencing reads were aligned to the human reference genome (GRCh37) using the BWA-MEM (v.0.7.17) algorithm. Post-alignment processing, including marking of duplicate reads and base quality score recalibration (BQSR), was performed according to the GATK (v.4.2.6.0) Best Practices workflow with default parameters. SNVs and small INDELs were identified using GATK HaplotypeCaller. Only variants located in the coding regions and exon boundary +/−20bp were selected. Variants that passed the GATK CNNScoreVariant 1D filter (FILTER = PASS) or fell within the Tranche 99.90–100.00 range were further filtered to retain variants with a variant allele frequency (VAF) of at least 20% and a sequencing depth of at least 20 [6]. This VAF threshold was selected to exclude recurrent low-level variants that were identified as mapping artifacts based on their consistent VAF values and clustered genomic positions across multiple unrelated samples. Variants with a minor allele frequency (MAF) greater than 0.1% in population databases (gnomAD) were considered non-pathogenic and excluded from further analysis. To further remove common ethnicity-specific polymorphisms, variants with MAF > 5% in the Korean Reference Genome Database (KRGDB) [7], a whole-genome sequencing database of 1722 Korean individuals, were also excluded. Recurrent variants of unknown significance (VUS) that persisted at high frequency despite 0.1% MAF filtering, often due to mapping artifacts misassigning reads to incorrect genomic loci, were excluded using a >10% threshold derived from the highest MAF (3.5%) in hereditary cancer cohorts and expanded three-fold for conservative filtering [8]. CNVs were detected using CNVkit (v.0.9.10) with a pooled normal reference constructed from 30 randomly selected blood samples from cancer patients within the cohort, using the CNVkit control construction algorithm based on median coverage across samples. The bin size was determined using the autobin option and segmentation was performed using the circular binary segmentation (CBS) algorithm. Copy number was derived as CN = 2 × 2^(log2 ratio)^, with loss defined as CN < 1.5 (log2 ratio < −0.4) and gain defined as CN ≥ 2.5 (log2 ratio > 0.3). The pathogenicity of variants was assessed according to the 2015 American College of Medical Genetics and Genomics (ACMG) and Association for Molecular Pathology (AMP) guidelines, with additional manual curation based on the ClinVar database (https://www.ncbi.nlm.nih.gov/clinvar/; accessed 22 December 2025), gene-specific evidence, and relevant literature review [9,10]. Variant validation using orthogonal methods such as Sanger sequencing was not performed in this study.

### 2.3 Data Analysis

Clinical characteristics were compared between patients with and without P/LP germline variants. Categorical variables were compared using Fisher’s exact test, and continuous variables were compared using the Mann-Whitney U test. The 95% confidence intervals for proportions were calculated using the Clopper-Pearson exact method. All statistical analyses were two-sided, with *p* < 0.05 considered statistically significant, and were performed using R software (version 4.1.2).

## 3 Results

A total of 108 patients were included in this study. Patient characteristics are demonstrated in Table 1. The median age was 9.0 years (range, 0.1–24.7 years) with male predominance (60.2%). The diagnoses were as follows: 31 medulloblastomas (MBLs), 25 intracranial germ cell tumors (iGCTs, 13 non-germinomatous germ cell tumors and 12 pure germinoma), 16 low-grade gliomas (LGGs), 16 high-grade gliomas (HGGs), 9 ependymomas (EPN), and 11 others. The median follow-up duration was 5.8 years (range, 0.6–13.2 years). Five patients had a history of multiple malignancies. Among them, three developed secondary cancers following treatment for primary CNS tumors: one patient with ependymoma later developed renal cell carcinoma, and two patients with MBL subsequently developed HGG. The remaining two developed CNS tumors after therapy for other malignancies: one with acute lymphoblastic leukemia (ALL) who later developed HGG, and another with rhabdomyosarcoma (RMS) who subsequently developed acute myeloid leukemia (AML) followed by MBL.

**Table 1:** Patient characteristics (*n* = 108).

Characteristic	Total (*n* = 108)	Patients with P/LP (*n* = 17), *n* (%, 95% CI)
**Male:Female, *n*:*n***	65: 43	11:6
**Age at diagnosis, median (range), years**	9.0 (0.1–24.7)	7.7 (1.0–24.0)
**Follow-up duration, median (range), years**	5.8 (0.6–13.2)	7.2 (0.8–13.2)
**Diagnosis**		
MBL, *n*	31	3 (9.7, 3.3–24.9)
LGG, *n*	16	5 (31.3, 14.2–45.6)
HGG, *n*	16	4 (25.0, 10.2–45.5)
EPN, *n*	9	0 (0, 0–29.9)
iGCT, *n*	25 (NGGCT 13, germinoma 12)	1 (4.0, 0.7–19.5)
Others, *n*	11 (3 ATRT, 2 PNET*, 2 pineoblastoma, 3 glioneuronal tumor, and 1 schwannoma)	4 (36.4, 15.2–54.6)
**Multiple cancer history, *n***	5	3 (60, 23.1–75.2)

Abbreviations: ATRT, atypical teratoid rhabdoid tumor; EPN, ependymoma; GCT, germ cell tumor; HGG, high grade glioma; LGG, low grade glioma; MBL, medulloblastoma; NGGCT, non-germinomatous germ cell tumor; P/LP, pathogenic/likely pathogenic; PNET, primitive neuroectodermal tumor. *PNET was classified according to the diagnostic criteria in use at the time of diagnosis; molecular data for reclassification under the WHO CNS5 framework were not available.

**Figure 1:**
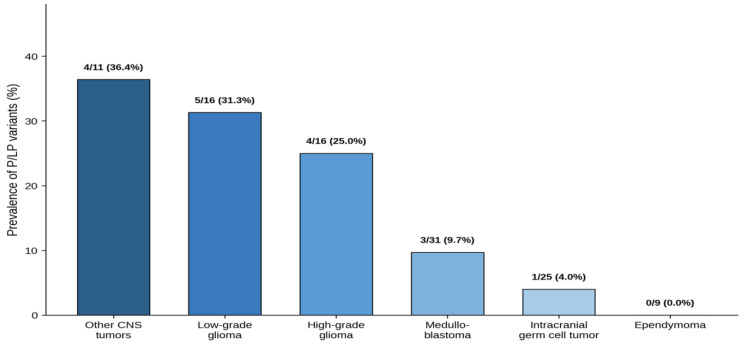
Prevalence of pathogenic/likely pathogenic (P/LP) germline variants by central nervous system (CNS) tumor subtype. The highest prevalence was observed in other CNS tumors (36.4%), followed by low-grade glioma (31.3%) and high-grade glioma (25.0%).

**Figure 2:**
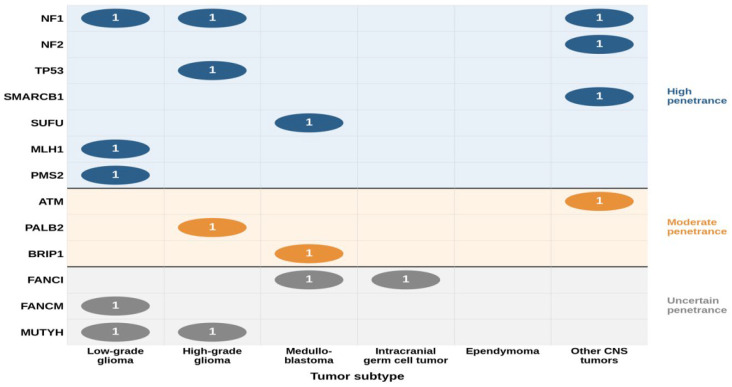
Gene–tumor landscape of pathogenic/likely pathogenic (P/LP) germline variants. Numbers within bubbles indicate the number of affected patients. Color coding reflects penetrance classification: high (blue), moderate (orange), and uncertain (gray).

The median on-target coverage depth was 1425× (range, 799×–2382×), with 99.70% of target bases covered at ≥100× and a mean coverage uniformity of 96.82%. A total of 695 germline variants were identified in 108 patients. P/LP variants were identified in 17 patients (15.7%, 95% CI: 10.1–23.8%), including four with a family history of cancer, suggesting potential hereditary predisposition. Among the 17 patients with P/LP variants, five (29.4%) had a previously established clinical diagnosis of a cancer predisposition syndrome (NF1, *n* = 3; NF2, *n* = 1; Li-Fraumeni syndrome, *n* = 1), and three (17.6%) were clinically suspected based on clinical features or family history. The remaining nine patients (52.9%) had no prior clinical suspicion of a germline predisposition. There were no significant differences in age at diagnosis (median 7.7 vs. 9.0 years, *p* = 0.50) or sex distribution (male-to-female ratio 11:6 vs. 65:43, *p* = 0.58) between patients with and without P/LP variants. Multiple malignancies were significantly more frequent in patients with P/LP variants than in those without (3/17 vs. 2/91, *p* = 0.03). The prevalence of P/LP variants differed significantly across tumor subtypes (*p* = 0.03), with the highest rates observed in other CNS tumors (4/11, 36.4%), followed by gliomas (9/32, 28.1%; 5 LGGs and 4 HGGs), and MBLs (3/31, 9.7%) (Fig. 1). Among patients with iGCTs, only one case (1/25, 4.0%) showed a P/LP germline variant. Patient characteristics with P/LP germline variants are listed in Table 2. The distribution of P/LP germline variants across genes and tumor types, along with gene-level penetrance classification, is illustrated in Fig. 2.

**Table 2:** Patient characteristics with pathogenic/likely pathogenic germline variants.

Pt. No.	Dx.	Age at Dx. (yr)	Sex	Gene	*Penetrance	Zygosity	Nucleotide Change	Amino Acid Change	ClinVar	Comment
1	MBL	2.8	F	SUFU	H	Hetero	c.268_270del	p.Tyr90del	-	*De novo* mutation (mother/father negative) Gorlin syndrome features including macrocephaly and frontal bossing
2	MBL	8.3	M	FANCI	U	Hetero	c.3352G>T	p.Glu1118Ter	LP	-
3	MBL	8.2	M	BRIP1	Mod	Hetero	c.2464dup	p.Tyr822LeufsTer10	P/LP	Multiple cancer history including rhabdomyosarcoma, acute myeloid leukemia and MBL
4	LGG	6.6	M	FANCM	U	Hetero	c.4515+1G>C	p.?	-	-
5	LGG	1.9	M	PMS2	H	Hetero	c.2620T>G	p.Ter874GlyextTer2	^†^Uncertain Significance^†^	-
6	LGG	1.0	M	MLH1	H	Hetero	c.2080G>T	p.Glu694Ter	P	FHx: gastric cancer (grandfather), liver cancer (uncle)
7	LGG	7.6	M	NF1	H	Hetero	c.3113+1G>A	p.?	P	-
8	LGG	5.5	M	MUTYH	U	Hetero	c.324_336del	p.Lys108AsnfsTer34	P	-
9	HGG (glioblastoma)	14.4	M	PALB2	Mod	Hetero	c.1050_1053del	p.Thr351ArgfsTer4	P	-
10	HGG (anaplastic astrocytoma)	22.4	M	NF1	H	Hetero	c.5902C>T	p.Arg1968Ter	P	-
11	HGG (anaplastic astrocytoma)	1.1	F	MUTYH	U	Hetero	c.1092_1093insCAGGC	p.Lys365GlnfsTer45	LP	-
12	HGG (glioblastoma)	7.7	F	TP53	H	Hetero	c.528C>A	p.Cys176Ter	P	FHx: rhabdomyosarcoma (sibling), Li-Fraumeni syndrome family
13	iGCT (pure germinoma)	24.0	M	FANCI	U	Hetero	c.2656_2657del	p.Thr886PhefsTer53	LP	-
14	ATRT	2.7	F	SMARCB1	H	Hetero	c.496dup	p.Leu166ProfsTer14	-	FHx: bladder rhabdoid tumor (sibling)
15	Schwannoma	15.3	M	NF2	H	Hetero	c.1156G>T	p.Glu386Ter	-	Known NF2 patient with bilateral vestibular schwannoma, spinal cord meningioma and multiple neurofibromas
16	Glioneuronal tumor	16.7	F	NF1	H	Hetero	c.2041C>T	p.Arg681Ter	P	NF1 father. Known NF1 patient with café-au-lait spot, cutaneous neurofibroma, and Lisch nodule
17	Glioneuronal tumor	9.4	F	ATM	Mod	Hetero	c.1402_1403del	p.Lys468GlufsTer18	P	-

Abbreviations: ATRT, atypical teratoid rhabdoid tumor; Dx, diagnosis; F, female; FHx, family history; H, high; iGCT, intracranial germ cell tumor; HGG, high-grade glioma; LGG, low-grade glioma; LP, likely pathogenic; M, male; MBL, medulloblastoma; Mod, moderate; P, pathogenic; U, uncertain. *Penetrance was classified as high (established pediatric cancer predisposition syndrome), moderate (moderate-penetrance cancer susceptibility gene), or uncertain (uncertain penetrance for CNS tumors in heterozygous carriers), based on Kratz et al. [11]. ^†^The PMS2 c.2620T>G (p.Ter874GlyextTer2) variant is classified as “Uncertain significance” in ClinVar but was reclassified as likely pathogenic based on internal ACMG/AMP assessment: stop-loss variant disrupting the normal termination codon (PVS1 at moderate strength), extremely rare in population databases (gnomAD MAX_AF = 6.52 × 10^−^^5^; PM2), predicted deleterious by *in silico* tools (PP3), and the patient’s diagnosis of childhood glioma, a tumor type associated with germline mismatch repair gene variants (PP4).

In other CNS tumors, four patients carried P/LP germline variants: two with glioneuronal tumors (NF1 and ATM), one with schwannoma (NF2), and one with ATRT (SMARCB1). Among the patients with glioneuronal tumors, one patient had a pathogenic truncating NF1 variant (c.2041C>T, p.Arg681Ter), while another carried a frameshift mutation in ATM (c.1402_1403del, p.Lys468GlufsTer18), both classified as pathogenic in ClinVar. The NF2 variant identified in the patient with schwannoma (c.1156G>T, p.Glu386Ter) was a nonsense mutation predicted to cause premature protein truncation and was classified as likely pathogenic. This patient was a known case of NF2, with bilateral vestibular schwannomas, spinal cord meningioma, and multiple neurofibromas. Finally, in the patient with ATRT, a SMARCB1 variant (c.496dup) was identified, predicted to cause a frameshift and consequent loss of function.

In gliomas, germline P/LP variants were detected in five patients with LGG and four with HGG. Among LGGs, variants were identified in FANCM, PMS2, NF1, MLH1, and MUTYH. The FANCM splice-donor variant (c.4515+1G>C) and the PMS2 stop-lost variant (c.2620T>G, p.Ter874GlyextTer2) are both predicted to disrupt normal protein function. The NF1 variant (c.3113+1G>A, p.?) represents a canonical splice-site alteration. The MLH1 variant (c.2080G>T, p.Glu694Ter) is a stop-gained mutation reported as pathogenic in ClinVar. The MUTYH variant was a frameshift mutation (c.324_336del, p.Lys108AsnfsTer34) reported as pathogenic in ClinVar. In HGGs, variants included a MUTYH frameshift mutation (c.1092_1093insCAGGC, p.Lys365GlnfsTer45), an NF1 nonsense mutation (c.5902C>T, p.Arg1968Ter), a PALB2 frameshift mutation (c.1050_1053del, p.Thr351ArgfsTer4), and a TP53 stop-gained mutation (c.528C>A, p.Cys176Ter). The PALB2, MUTYH, and TP53 variants are reported as pathogenic in ClinVar, and the patient with the TP53 variant belonged to a Li-Fraumeni family.

In MBLs, three patients were found to carry P/LP germline variants: one in SUFU, one in BRIP1, and one in FANCI. The SUFU variant was an in-frame deletion (c.268_270del, p.Tyr90del), identified *de novo* in a patient with Gorlin syndrome. The BRIP1 mutation (c.2464dup, p.Tyr822LeufsTer10) was identified in a patient with multiple prior malignancies, including RMS and AML, who subsequently developed MBL. The patient remains alive to date without recurrence or development of other malignancies. In addition, a patient with high-risk MBL harbored a FANCI stop-gained splice-region mutation (c.3352G>T, p.Glu1118Ter), reported as likely pathogenic in ClinVar.

Among the 25 patients with iGCTs, one patient was found to have a P/LP FANCI frameshift variant (c.2656_2657del, p.Thr886PhefsTer53). No P/LP variants were detected in the nine patients with ependymoma.

In addition to these P/LP variants, we identified three additional germline variants in patients with medulloblastoma that did not meet ACMG criteria for pathogenicity but may represent potentially relevant alterations. These were: (1) FGFR1 c.1444_1446del (p.Ser483del), an in-frame deletion within the kinase domain, found in a patient who later developed a HGG; (2) BRCA2 c.9275A>G (p.Tyr3092Cys), detected in a patient who carried the variant and whose two paternal cousins had also been diagnosed with MBL at the ages of 6 and 9 years, respectively, suggesting possible familial segregation; and (3) SUFU c.1028G>T (p.Arg343Leu), a missense variant in a well-established medulloblastoma predisposition gene. Both the BRCA2 and SUFU variants are currently classified as Conflicting in ClinVar.

## 4 Discussion

In this study, we investigated the prevalence and clinical implications of germline cancer predisposition gene variants in Korean children and AYAs with CNS tumors. We identified P/LP variants in 15.7% of patients, which is somewhat higher than the 8–10% prevalence previously reported in pediatric cancer patients [3,12,13] but consistent with CNS tumor-specific studies reporting rates of 8–23% [4,5]. Our study provides the first systematic characterization of germline predisposition in Korean children and AYA patients with CNS tumors, extending the existing literature which has been predominantly derived from Western cohorts. Notably, several P/LP variants identified in our cohort, including PMS2 c.2620T>G and FANCI c.2656_2657del, were absent in European populations in gnomAD [14], highlighting the potential importance of germline studies in diverse ethnic populations. Furthermore, over half of P/LP variants were detected in patients without prior clinical suspicion, reinforcing the value of systematic germline testing beyond phenotype-based approaches.

The spectrum of germline variants observed in our cohort was largely consistent with prior studies and provided new insights. SMARCB1 and SUFU variants were identified in expected tumor contexts, supporting the well-established role of these genes in CNS tumor predisposition [15,16]. Notably, NF1 emerged as the most frequently affected gene, identified across LGGs, HGGs, and a glioneuronal tumor, indicating that NF1 predisposition is not limited to tumor grade and may underlie diverse tumors within the glioma–glioneuronal spectrum [17]. All three NF1 variants reside upstream of or adjacent to the GAP-related domain (GRD), a critical region for neurofibromin’s RAS-GAP tumor-suppressor function [18], and are therefore expected to impair downstream RAS regulation.

Another significant observation was the recurrent involvement of DNA repair genes, including FANCI, FANCM, PMS2, BRIP1, MUTYH, and PALB2. These genes participate in major repair pathways, such as Fanconi anemia–associated repair, homologous recombination, base excision repair, and mismatch repair, suggesting that defects in these mechanisms may represent an underrecognized yet potential contributor to tumor predisposition in pediatric CNS tumors. This is consistent with emerging evidence implicating disruptions in these DNA repair pathways in pediatric brain tumor biology [19,20,21], including a recent international study of pediatric diffuse midline glioma reporting enrichment of germline variants in homologous recombination and Fanconi anemia pathway genes [22]. Of note, several of these genes including MUTYH, FANCI, and FANCM, are classically associated with autosomal recessive syndromes, and the pathogenic significance of the heterozygous variants identified in our cohort as drivers of CNS tumor predisposition remains to be determined.

In contrast, inherited predisposition appears to play a limited role in iGCTs and ependymomas. Most iGCTs are thought to arise from somatic events involving the KIT–RAS–MAPK signaling cascade rather than inherited susceptibility [23,24]. Previous molecular studies have not identified consistent germline alterations in canonical cancer predisposition genes [25,26]. Similarly, ependymomas show a low frequency of germline mutations, with fewer than 4% of pediatric cases attributable to genetic predisposition [27]. Our findings are consistent with these observations.

Beyond variants classified as P/LP, we identified additional germline alterations in established or putative predisposition genes, including an FGFR1 in-frame deletion, a BRCA2 missense variant, and a SUFU missense variant. FGFR1 alterations have been reported in pediatric LGGs, suggesting a potential role in MAPK pathway activation [28]. The patient carrying this variant developed multiple tumors, including MBL followed by HGG. While BRCA2 is primarily associated with breast and ovarian cancer susceptibility, it has also been reported in rare families with pediatric brain tumors, including MBL, suggesting a possible broader role in cancer predisposition [15,29]. In our cohort, the patient carried the BRCA2 c.9275A>G (p.Tyr3092Cys) variant, and her two paternal cousins had also been diagnosed with MBL. While the current classification in ClinVar remains “conflicting”, the variant lies within the C-terminal DNA-binding domain, within the OB3 fold region critical for DNA interaction and RAD51 filament stabilization, indicating potential functional significance. SUFU is a well-established MBL predisposition gene, and while most reported pathogenic variants are truncating, missense variants may also confer risk. SUFU functions as a key negative regulator of Hedgehog signaling via GLI binding, and amino acid substitutions affecting these domains can impair its tumor suppressor activity [30,31]. Germline SUFU missense variants have indeed been reported in patients with MBL or Gorlin syndrome [32,33], suggesting that such variants, though yet classified as pathogenic, warrant further evaluation. To clarify their significance, additional studies are needed, including functional assays of protein activity, segregation analyses in affected families, and tumor-based analyses such as loss of heterozygosity or second-hit events.

From a clinical management perspective, germline findings may influence several aspects of care. First, at the diagnostic stage, recognition of a germline predisposition prompts systematic assessment for subtle syndromic features, targeted evaluation for additional associated lesions, and early referral for genetic counseling. This is especially relevant for genes such as NF1, SMARCB1, SUFU, and mismatch-repair genes, where accurate diagnosis of the underlying syndrome directly informs anticipatory care. Second, surveillance strategies can be substantially modified based on germline status. For example, children with NF1 or NF2 require scheduled screening for optic pathway gliomas, spinal tumors, or schwannomas; patients with SUFU-related medulloblastoma predisposition benefit from early-childhood brain magnetic resonance imaging (MRI) surveillance; and carriers of DNA repair gene defects (e.g., MLH1, PMS2, BRIP1, PALB2) may require extracranial cancer screening following established syndrome-specific protocols [34,35,36]. In our cohort, all patients with P/LP variants were referred for genetic counseling, and syndrome-specific surveillance was initiated accordingly. Finally, germline information can also affect therapeutic approaches. In our cohort, a radiation-sparing approach was adopted for the patient with a TP53 variant, and the patient with SUFU-related Gorlin syndrome was treated without radiation. For NF1-associated gliomas, targeted agents such as selumetinib have been considered in cases with refractory disease, and bevacizumab was administered for the patient with NF2-related schwannoma. Beyond these established management implications, germline findings may also inform emerging therapeutic strategies. Tumors arising in the setting of homologous recombination deficiency may be candidates for poly ADP-ribose polymerase (PARP) inhibition in selected contexts [37,38]. In addition, mismatch repair–deficient gliomas may respond differently to alkylating agents and exhibit susceptibility to immune checkpoint inhibition, but response is heterogeneous and influenced by molecular subtype [37,39,40]. However, no somatic second-hit analysis or functional validation was performed in this study, and whether these germline findings translate into actionable therapeutic targets would require further somatic and functional characterization. Importantly, over half of the patients with P/LP variants in our cohort lacked a family history or overt syndromic features, highlighting the limitations of phenotype-based testing and supporting broader integration of germline evaluation in pediatric CNS tumor care.

While these findings suggest meaningful clinical implications, they should be interpreted with caution given the exploratory nature of this study. Strengths of this study include the use of a comprehensive pediatric cancer gene panel, detailed clinic-genomic correlation, and standardized ACMG/AMP variant classification. However, this study is limited by its single-center retrospective design at a referral center, which may overestimate predisposition frequency, as well as its modest cohort size. As the cohort includes patients diagnosed over a wide time span, integrated molecular classification according to the WHO 2021 criteria was not uniformly available, and tumor diagnoses are based primarily on histopathological classification. The absence of functional validation, orthogonal CNV confirmation, or systematic familial segregation analysis is another limitation. Although *de novo* occurrences were confirmed in selected cases (e.g., SUFU), parental testing was not routinely performed for all identified variants, which limits definitive determination of inheritance patterns and may affect pathogenicity interpretation for certain variants. In addition, family history data were more thoroughly collected in P/LP carriers through genetic counseling than in non-carriers, limiting direct comparison of family history between the two groups. These preliminary observations therefore require confirmation in larger, multicenter cohorts with systematic familial segregation testing, paired germline–somatic profiling, and functional validation.

## 5 Conclusion

In this cohort, germline P/LP variants were identified in 15.7% of Korean children and AYAs with CNS tumors, occurring most frequently in gliomas and other CNS tumors. These findings highlight the contribution of hereditary predisposition to specific CNS tumor subtypes and support the incorporation of germline testing into routine clinical practice to refine diagnosis, guide therapy, and inform genetic counseling. Early germline evaluation and tailored surveillance strategies may help optimize long-term outcomes in this population.

## Data Availability

The data supporting the findings of this study are available from the corresponding author upon reasonable request.

## Supplementary Materials

The supplementary material is available online at https://www.techscience.com/doi/10.32604/or.2026.079120/s1.
