# Supplementary material for: Germline Predisposition in Pediatric Central Nervous System Tumors: Insights from a Multigene Panel Study
Source: Oncol Res. 2026 May 21;34(6):20. doi: 10.32604/or.2026.079120 (PMC13223255; doi:10.32604/or.2026.079120)
Supplement: Supplementary file 1 [file OncolRes-34-79120-s001.zip › TSP_OR_79120-s001.docx]

Supplementary Table S1. Gene panel list

| *MET* | *ATIC* | *CCND1* | *DDR2* | *EXT2* | *GAB1* | *ITK* | *MTHFR* | *NUDT15* | *PRF1* | *SDHC* | *TCF12* |
| --- | --- | --- | --- | --- | --- | --- | --- | --- | --- | --- | --- |
| *TERT* | *ATM* | *CCND2* | *DDX3X* | *EZH2* | *GATA1* | *JAK1* | *MTOR* | *ODZ3* | *PRKAR1A* | *SDHD* | *TET2* |
| *ALK* | *ATR* | *CCND3* | *DICER1* | *FAH* | *GATA2* | *JAK2* | *MTRR* | *OR5T1* | *PTCH1* | *SEMA3C* | *TFE3* |
| *EWSR1* | *ATRX* | *CD79A* | *DIS3L2* | *FANCA* | *GATA3* | *JAK3* | *MUC16* | *OTX2* | *PTCH2* | *SERPINB3* | *TGFBR1* |
| *RET* | *AURKA* | *CD79B* | *DNMT3A* | *FANCB* | *GFI1B* | *KDM6A* | *MUC4* | *PALB2* | *PTEN* | *SETBP1* | *TGFBR2* |
| *ROS1* | *AURKB* | *CDC73* | *DPYD* | *FANCC* | *GLI1* | *KDR* | *MUTYH* | *PARP1* | *PTPN11* | *SETD2* | *TINF2* |
| *FOXO1* | *AXIN1* | *CDH1* | *DROSHA* | *FANCD2* | *GLI2* | *KIF1B* | *MYB* | *PAX5* | *PTPRD* | *SF3B1* | *TMEM127* |
| *RELA* | *AXIN2* | *CDK4* | *DUSP10* | *FANCE* | *GNA11* | *KIT* | *MYC* | *PBRM1* | *RAB27A* | *SH2B3* | *TP53* |
| *SS18* | *B2M* | *CDK6* | *EBF1* | *FANCF* | *GNAQ* | *KMT2A* | *MYCL* | *PDE6G* | *RAC1* | *SHH* | *TPMT* |
| *ABCB1* | *BAP1* | *CDK8* | *EGFR* | *FANCG* | *GNAS* | *KMT2C* | *MYCN* | *PDGFRA* | *RAD21* | *SHOC2* | *TRIM37* |
| *ABCB11* | *BARD1* | *CDKN1C* | *EIF3H* | *FANCI* | *GPC3* | *KMT2D* | *MYD88* | *PDGFRB* | *RAD50* | *SHROOM2* | *TSC1* |
| *ABCC1* | *BCL2* | *CDKN2A* | *EML4* | *FANCL* | *GPC4* | *KRAS* | *NBN* | *PDPK1* | *RAD51C* | *SLCO1B1* | *TSC2* |
| *ABCC3* | *BCL6* | *CDKN2B* | *EP300* | *FANCM* | *GREM1* | *LAMA5* | *NF1* | *PHF6* | *RAD51D* | *SMAD4* | *TTN* |
| *ABCG2* | *BCOR* | *CDKN2C* | *EPCAM* | *FAT1* | *GSTM1* | *LMO1* | *NF2* | *PHOX2B* | *RAF1* | *SMAD7* | *TXNDC15* |
| *ABL1* | *BCORL1* | *CEBPA* | *EPHA3* | *FBXW7* | *GSTP1* | *LRP1B* | *NFE2L2* | *PIK3CA* | *RB1* | *SMARCA1* | *TXNDC16* |
| *ACVR1* | *BLM* | *CEP57* | *EPHB1* | *FGFR1* | *H3F3A* | *MAP2K1* | *NFKBIA* | *PIK3CB* | *RECQL4* | *SMARCA4* | *TYK2* |
| *AHNAK2* | *BMP4* | *CHEK1* | *EPHB4* | *FGFR2* | *HDAC2* | *MAP2K2* | *NHP2* | *PIK3R1* | *REST* | *SMARCB1* | *U2AF1* |
| *AIP* | *BMPR1A* | *CHEK2* | *ERBB2* | *FGFR3* | *HIF1A* | *MAX* | *NKX2-1* | *PIK3R2* | *RHBDF2* | *SMO* | *UGT1A1* |
| *AKT1* | *BRAF* | *CIC* | *ERBB3* | *FGFR4* | *HIST1H3B* | *MDM2* | *NOP10* | *PINK1* | *RHPN2* | *SNCAIP* | *VHL* |
| *AKT2* | *BRCA1* | *COLCA2* | *ERBB4* | *FH* | *HMBS* | *MDM4* | *NOTCH1* | *PML* | *RICTOR* | *SOD2* | *WAS* |
| *AKT3* | *BRCA2* | *CREBBP* | *ERCC1* | *FLCN* | *HNF1A* | *MED12* | *NOTCH3* | *PMS1* | *RIT1* | *SOS1* | *WRN* |
| *AMER1* | *BRIP1* | *CSF1R* | *ERCC2* | *FLG* | *HRAS* | *MEN1* | *NPAT* | *PMS2* | *RNF213* | *SPRED1* | *WT1* |
| *ANKRD26* | *BTK* | *CSF3R* | *ERCC3* | *FLI1* | *IDH1* | *MITF* | *NPM1* | *POLD1* | *RRAS2* | *SPRTN* | *XPA* |
| *APC* | *BUB1B* | *CSMD1* | *ERCC4* | *FLT3* | *IDH2* | *MLH1* | *NRAS* | *POLD3* | *RTEL1* | *SRC* | *XPC* |
| *APOBEC3A* | *C11ORF95* | *CTDNEP1* | *ERCC5* | *FOXL2* | *IDO1* | *MN1* | *NSD1* | *POLE* | *RUNX1* | *SRSF2* | *XRCC1* |
| *APOBEC3B* | *C8ORF34* | *CTNNB1* | *ERCC6* | *FOXR2* | *IGF1* | *MPL* | *NT5C2* | *POT1* | *RYR1* | *STAG2* | *XRCC3* |
| *ARID1A* | *CALR* | *CTR9* | *ERG* | *FRS2* | *IGF1R* | *MRE11A* | *NTHL1* | *POU6F2* | *SBDS* | *STAT3* | *YAP1* |
| *ARID1B* | *CARD11* | *CYLD* | *ESR1* | *FUBP1* | *IGF2R* | *MSH2* | *NTRK1* | *PPM1D* | *SDHA* | *STK11* | *ZNRF3* |
| *ARID2* | *CBL* | *DDB1* | *ETV6* | *G6PC* | *IKZF1* | *MSH3* | *NTRK2* | *PPOX* | *SDHAF2* | *SUFU* |  |
| *ASXL1* | *CBLB* | *DDB2* | *EXT1* | *G6PD* | *IL7R* | *MSH6* | *NTRK3* | *PRCC* | *SDHB* | *SUZ12* |  |
